# Cognitive overload in multimedia language learning: a review of attention limits and instructional design strategies

**DOI:** 10.3389/fpsyg.2026.1850074

**Published:** 2026-07-29

**Authors:** Yan Qi

**Affiliations:** Education, School of Foreign Languages, Taiyuan University of Science and Technology, Taiyuan, Shanxi, China

**Keywords:** artificial intelligence, cognitive load theory, cognitive overload, English as a foreign language, English as a second language, multimedia learning, speaking anxiety, technology-enhanced learning

## Abstract

**Background:**

Cognitive overload and speaking anxiety are major barriers to second language acquisition, particularly in English as a Foreign Language (EFL) and English as a Second Language (ESL) contexts. Multimedia learning, artificial intelligence (AI), mobile learning (m-learning), and cognitive-affective interventions have been proposed to enhance language performance and reduce anxiety; however, their overall effectiveness remains unclear.

**Objective:**

To evaluate the effects of multimedia-assisted language learning, cognitive-affective interventions, learner-related factors, and speaking anxiety across educational settings and to quantify their effectiveness through meta-analysis.

**Methods:**

This meta-analysis followed systematic evidence-synthesis procedures. A total of 38 studies published between 2013 and 2026 were included. Risk of bias was assessed using the ROBINS-I tool, and evidence quality was evaluated using GRADE criteria. Random-effects inverse-variance models were applied to calculate standardized mean differences (SMDs) with 95% confidence intervals (CIs). Heterogeneity was assessed using the I^2^ statistic. Publication bias was examined through funnel plots and Egger’s regression test, while robustness was evaluated using leave-one-out sensitivity analyses.

**Results:**

Significant positive effects were observed across all four domains (*p* < 0.05). Multimedia and technology-enhanced language learning demonstrated a pooled SMD of 0.44 (95% CI: 0.31–0.58; *I*^2^ = 45.6%). Anxiety-reduction and cognitive-affective interventions showed an SMD of 0.51 (95% CI: 0.40–0.62; *I*^2^ = 13.5%). Studies examining anxiety, academic achievement, and learner-related factors yielded an SMD of 0.51 (95% CI: 0.41–0.62; *I*^2^ = 27.5%). The strongest effect was observed for interventions targeting speaking anxiety in educational settings (SMD = 0.54, 95% CI: 0.47–0.62; *I*^2^ = 16.7%). ROBINS-I assessments indicated predominantly moderate risk of bias, whereas GRADE ratings ranged from high certainty for multimedia-based interventions to low-to-moderate certainty for context-specific approaches. Egger’s test detected no significant publication bias (*p* > 0.05). Sensitivity analyses confirmed the stability of pooled estimates, with effect sizes ranging from 0.52 to 0.54.

**Conclusion:**

Multimedia-assisted learning, AI-enhanced educational environments, and cognitive-affective interventions significantly improve language learning outcomes and reduce speaking anxiety. Speaking anxiety remains a key determinant of language achievement, while psychological factors such as self-efficacy and confidence play crucial roles in successful language acquisition.

## Introduction

Multimedia innovations, including instructional formats such as video-based lessons, narrated presentations, mobile applications, and adaptable digital platforms, are now widely used in language acquisition. When properly developed, these methods enable the simultaneous presentation of verbal and visual information, which can aid vocabulary development and language comprehension. However, integrating multiple information sources also places learners under greater cognitive strain, particularly in terms of processing power and attentional control (Huang et al., 2025; Li et al., 2025). The main theoretical framework for comprehending these learning processes is provided by Cognitive Load Theory (CLT) and Cognitive Theory of Multimedia Learning (CTML) (Lv et al., 2024; Lv et al., 2024; Yin et al., 2024). According to CLT, learning is restricted by working memory capacity, and learning outcomes are influenced by three complimentary types of cognitive load (Lv et al., 2024; Lv et al., 2024).

The intrinsic complexity of language-learning tasks, such as vocabulary acquisition, reading and listening comprehension, speaking activities, learner proficiency, and the processing demands related to EFL and ESL instruction, was operationalised as intrinsic cognitive load in this review. The presentation and integration of text, audio, images, captions, subtitles, e-notes, mobile learning applications, artificial intelligence-supported instruction, augmented reality, and other multimedia interfaces were all considered forms of extraneous cognitive load (Lv et al., 2024; Lv et al., 2024). Germane cognitive load was defined as the productive cognitive effort required for successful language learning, including schema development, vocabulary retention, understanding, feedback processing, and the integration of verbal and visual information (Lv et al., 2024; Lv et al., 2024).

CTML complements CLT by clarifying that learners receive verbal and visual information through distinct but capacity-limited channels and that good coordination of these channels during multimedia instruction is necessary for meaningful learning (Yin et al., 2024). Therefore, whether multimedia instructional approaches handled inherent task complexity, reduced needless processing, and encouraged productive cognitive processing was the basis for interpreting the findings compiled in this review. This theoretical framework also helped to organise the review by distinguishing direct research on multimedia learning and cognitive load from related evidence on cognitive-affective interventions, learner-related factors, and speaking anxiety in EFL/ESL language-learning environments.

Learners frequently have to analyze text, auditory, and visual inputs simultaneously in multimedia language learning environments, which can cause cognitive overload if teaching materials are poorly organized. This review’s empirical research shows that instructional strategies such as content segmentation can reduce cognitive load and enhance vocabulary acquisition, retention, and reading comprehension (Lv et al., 2024). On the other hand, differences in attention and recall performance have been linked to higher visual load and inefficient presentation forms, underscoring the significance of controlling cognitive demands (Lv et al., 2024; Yin et al., 2024).

In multimedia learning environments, attention is a key factor in managing cognitive load. Learners must filter out irrelevant information and devote their limited attentional resources to pertinent aspects of instruction. Increased processing demands can adversely affect recall, especially when many stimuli compete for attention, according to experimental data from audiovisual tasks (Cao et al., 2025). In a similar vein, it has been demonstrated that multimedia lecture formats with speech-enabled translation affect learner engagement, attention, and cognitive load, illustrating the relationship between instructional design and cognitive processing (Sun et al., 2025; Wang et al., 2026; Feng et al., 2025).

Language learning research has examined a variety of multimedia teaching strategies that represent different approaches to managing cognitive load. For example, it has been demonstrated that code-switching interfaces with native language support help ESL learners acquire vocabulary (Lin et al., 2026; Qiao et al., 2026). Variations in cognitive load and learning performance have also been linked to the use of subtitles and taking notes in multimedia contexts (Li et al., 2026; Chu et al., 2025; Qiao et al., 2026). Furthermore, engagement, achievement, and cognitive load have all been examined in relation to mobile-based language learning applications; the results suggest that cognitive load may function as a mediating factor in learning outcomes (Zhang and Chen, 2026). These tactics are further expanded by AI-assisted learning systems and adaptive teaching methods, which offer customized learning paths and feedback systems (Tu et al., 2026; Yan et al., 2026; Jiang et al., 2023).

Multimodal and emergent technologies are being investigated in response to advances in multimedia learning. The balance between the advantages of dual coding and the cognitive burden in vocabulary learning has been investigated in studies examining multimodal input settings (Li et al., 2022). In a similar vein, the effects of augmented reality-enhanced learning environments on learning efficacy, motivation, and cognitive load under various teaching circumstances have been assessed (Siregar, 2022). These results highlight how crucial it is to match multimedia design elements to learners’ cognitive abilities.

These research assessments of cognitive strain are multifaceted. The majority of studies used subjective measures, such as standardized cognitive load scales and self-report tools, alongside performance-based outcomes, including comprehension, recall, and vocabulary acquisition. Furthermore, objective techniques such as electroencephalography have been employed to measure cognitive load in real time during multimedia tasks, providing supplementary information about cognitive processes (Simanungkalit and Likuayang, 2023).

The relationship between cognitive load and learner-related factors, such as language proficiency, learning styles, and psychological variables, has also been investigated. Differences in perceived cognitive load and learning performance in multimedia contexts have been linked to different learning styles (Lin et al., 2016). Furthermore, cognitive load, engagement, and anxiety have been linked in studies examining language learning environments, particularly in mobile-based and classroom settings (Simanungkalit and Likuayang, 2023). To critically examine the effect of multimedia-assisted language learning, cognitive and affective treatments, learner variables, and speaking anxiety in different educational settings, and to establish the size and reliability of their effects using meta-analysis.

## Methodology

### Study design

This investigation is a systematic review and meta-analysis of the effects of multimedia-assisted language learning, cognitive-affective interventions, speaking anxiety, and learner variables on language learning outcomes among EFL and ESL learners. The study was conducted in accordance with established procedures for evidence synthesis and quantitative meta-analysis.

### Search strategies

An extensive literature search was conducted in important electronic databases, including PubMed, Embase, Cochrane Library, Web of Science, and Google Scholar. Research papers from January 2013 to March 2026 were considered. The search terms used include: “Multimedia Learning,” “Cognitive Load,” “Technology-Enhanced Language Learning,” “Artificial Intelligence,” “Mobile-Assisted Language Learning,” “Speaking Anxiety,” “Foreign Language Anxiety,” “English Language Learning,” “Self-Efficacy,” and “Language Performance”(Table 1).

**Table 1:** Literature review strategies.

Search engine	Date range	Keywords	Filters applied
PubMed	January 2013—March 2026	Multimedia learning, cognitive load, technology-enhanced language learning, artificial intelligence, mobile-assisted language learning, speaking anxiety, foreign language anxiety, English language learning, self-efficacy, language performance	Experimental studies, survey-based studies, cross-sectional studies, peer-reviewed articles, English language, full text available
Embase	January 2013—March 2026	Multimedia learning, cognitive load, technology-enhanced learning, AI in education, language anxiety, language performance	Experimental research, observational surveys, cross-sectional designs, journal articles, English language
Cochrane library	January 2013—March 2026	Cognitive load, multimedia learning, language learning interventions, educational technology	Randomized controlled trials, experimental studies, systematic reviews with included experimental evidence, English language
Web of science	January 2013—March 2026	Multimedia learning, AI-based learning, mobile-assisted language learning, foreign language anxiety, self-efficacy	Experimental studies, survey research, cross-sectional studies, peer-reviewed indexed journals, citation-tracked articles, English language
Google scholar	January 2013—March 2026	Multimedia learning, cognitive load, AI in language learning, speaking anxiety, language performance, technology-enhanced learning	Experimental studies, survey-based studies, cross-sectional studies, peer-reviewed and grey literature, English language, full-text availability

### Inclusion criteria

Those studies that examined: (1) EFL or ESL students; (2) multimedia learning, computer-mediated teaching, cognitive-affective techniques, and anxiety of speaking; (3) quantitative results concerning language learning, anxiety, achievement and learner variables; (4) enough statistical information to compute effect sizes; and (5) which appeared in peer-reviewed journals or academic dissertations written in English were included.

### Exclusion criteria

Those studies that did not contain: (1) appropriate outcome measures; (2) adequate statistical information; (3) abstracts, editorials, and review articles; and (4) samples unrelated to language learning were excluded from the analysis.

### Study selection and data extraction

Screening of titles and abstracts was conducted independently, followed by full-text screening of potentially eligible studies. The data extracted included author(s), year of publication, country of research, study design, participant information, educational status, intervention details, number of participants, outcomes, and statistics.

### Quality assessment

The methodological quality assessment was conducted using the ROBINS-I (Risk of Bias in Non-randomized Studies of Interventions) tool. The seven domains used for this purpose included confounding, participant selection, intervention characterization, deviations from intended interventions, missing data, outcome measurement, and selective reporting. The overall risk of bias was categorized as low, low-to-moderate, moderate, or serious. Certainty of evidence was assessed based on the GRADE (Grading of Recommendations, Assessment, Development, and Evaluation) criteria.

### Synthesis of data and statistical analysis

A meta-analysis was conducted using a random-effects model with the inverse-variance technique due to heterogeneity among studies. Standardized Mean Difference (SMD) with 95% Confidence Intervals (CIs) was determined as the effect size estimate.

Multimedia and technology-enhanced language acquisition; anxiety reduction and cognitive-affective therapies; anxiety, academic achievement, and learner factors; and speaking anxiety across educational contexts were the four distinct areas into which research were divided for synthesis. The direct multimedia and cognitive-load evidence base was represented by the first domain, while associated cognitive-affective and learner-level data was represented by the other domains. Therefore, domain-specific estimations were interpreted independently in order to avoid using correlational learner factors or speaking anxiety as direct indicators of cognitive load.

Statistical heterogeneity among studies was assessed using the Q statistic and the *I*^2^ statistic, with low, moderate, and high heterogeneity defined as *I*^2^ values of 25, 50, and 75%, respectively. Variability across studies was quantified by using τ^2^.

The existence of publication bias was assessed by funnel plot inspection and Egger’s regression test. The sensitivity analysis was performed using the “leave-one-out” method, in which the effect of excluding individual studies was assessed.

### Outcome measures

The main outcomes were language-learning effectiveness, cognitive load, anxiety about speaking, foreign-language anxiety, and academic success. The secondary outcomes were self-confidence, self-efficacy, learner involvement, motivation, attitude towards language learning, communicative competence, and psychological factors affecting language acquisition.

## Results

### Study selection

**Figure 1:**
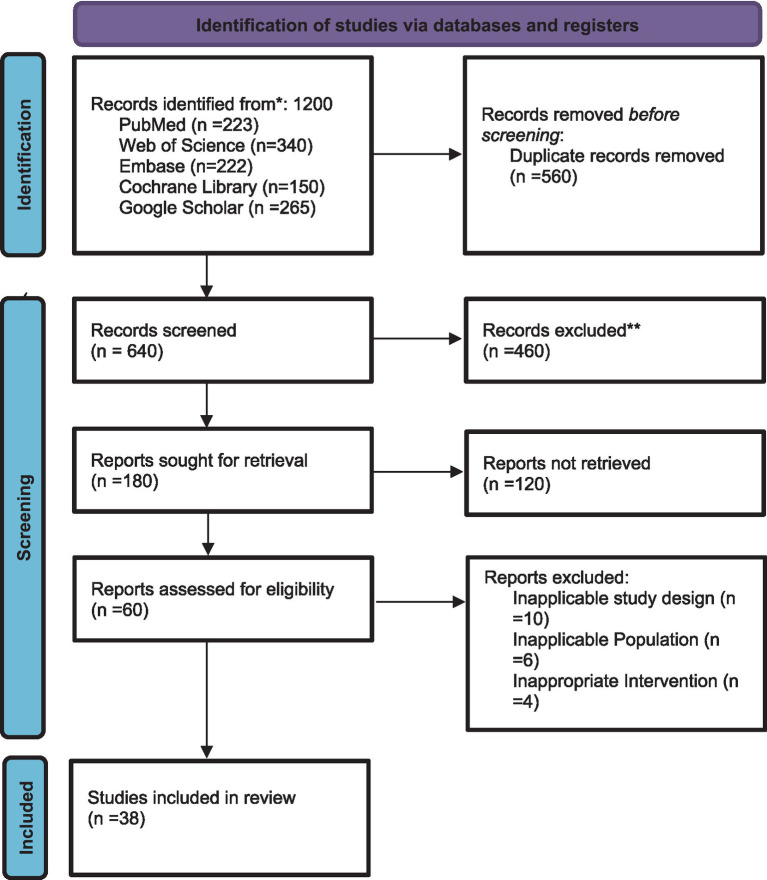
PRISMA flow chart of study selection.

The researchers conducted a systematic search of major databases, including PubMed, Embase, Cochrane Library, Web of Science, and Google Scholar, to identify studies examining cognitive overload in multimedia language learning. The search results showed 1,200 articles that could potentially match the research criteria. The research team evaluated 180 studies for eligibility after removing duplicate studies and examining titles and abstracts against three specific inclusion criteria: peer-reviewed publication, use of multimedia interventions in language learning, and assessment of cognitive load or related learning outcomes. The 38 included studies met all required inclusion criteria for the final analysis (Figure 1).

### Baseline characteristics of the included studies

A total of 38 articles published between 2013 and 2026 have been reviewed, with studies conducted in various locations, including Indonesia, Malaysia, China, Taiwan, Thailand, Turkey, the Philippines, Pakistan, and the United States. The majority of studies employed cross-sectional or correlational designs, although some also used experimental, case study, and mixed-methods designs. Study participants varied widely, ranging from elementary to university-level students, with EFL/ESL learners forming the bulk of the sample and representing diverse language abilities. Instructional settings involved multimedia learning, mobile and AI learning tools, AR applications, and foreign language anxiety intervention programs (Table 2).

**Table 2:** Baseline characteristics of the included studies.

Study	Country	Study design	Education level	Language proficiency	Instructional design features	Primary outcome	Secondary outcomes
(Mayer et al., 2014)	USA	Experimental	Undergraduate	Non-native English	Video vs. captions	Comprehension scores	Cognitive load implication
(Puri et al., 2025)	China	Cross-sectional survey	University	Chinese language learners	Mobile tool engagement	Speaking and learning anxiety	Engagement and achievement
(Lin et al., 2016)	Taiwan	Experimental (2 × 2 factorial)	Undergraduate	EFL learners	Subtitles and enotes vs. none	Load reduction w/subtitles	Performance differences
(Li et al., 2022)	China	Experimental	Universithe ty	EFL learners	Dual coding vs. cognitive load conditions	Effect on vocab learning	Vocabulary outcomes
(Feng et al. 2025)	China	Experimental (pretest–posttest)	Undergrad	English L2	Personalized feedback, adaptive learning, speech recognition, and intelligent tutoring	EFL learning outcomes	Learning performance comparisons
(Chen et al., 2022)	Taiwan	Experimental	Undergrad	EFL learners	Captions and proficiency conditions	EFL learning outcomes	Motivation and attitude
(Tam, 2026)	China	Mixed-methods/empirical	Higher ed	Not specified	Multimodal feedback	Student perceptions	Practices of multimodal feedback
(Sinadia and Ngingi, 2023)	Indonesia	Survey	Secondary	EFL learners	Vocational English speaking tasks	Sources of speaking anxiety	Student perceptions
(Marpaung and Fithriani, 2023)	Indonesia	Cross-sectional survey	Junior high school students	EFL learners	Investigation of factors contributing to English-speaking anxiety and potential coping strategies	English-speaking anxiety	Causes of anxiety; suggested anxiety-reduction strategies
(James et al., 2019)	Malaysia	Case study	Undergraduate students	ESL learners	Exploration of anxiety experiences in English language learning contexts	English language anxiety	Sources of anxiety: impact on classroom participation and language performance
(Zamri and Hashim, 2023)	Malaysia	Cross-sectional correlational study	Undergraduate students	ESL learners	Assessment of the relationship between speaking anxiety and English proficiency	Speaking anxiety and English proficiency	Correlation between anxiety and proficiency levels
(Miskam and Saidalvi, 2019)	Malaysia	Cross-sectional survey	Undergraduate students	ESL learners	Assessment of English-speaking anxiety among university learners	English-speaking anxiety	Factors influencing anxiety and communication performance
(Keny et al., 2026)	Indonesia	Cross-sectional survey	Undergraduate students	EFL learners	Examination of language anxiety factors affecting self-confidence in English-speaking	Language anxiety and speaking self-confidence	Relationship between anxiety dimensions and confidence
(Badriyah and Novita, 2023)	Indonesia	Cross-sectional survey	Junior high school students	EFL learners	Investigation of anxiety in English-speaking classes	English-speaking class anxiety	Factors contributing to anxiety in classroom settings
(Said and Weda, 2018)	Indonesia	Case study	University students	EFL learners	Assessment of English language anxiety and its impact on oral communication	English language anxiety	Effects on oral communication skills and classroom participation
(Tamco, 2021)	Philippines	Cross-sectional survey	Senior high school students	EFL learners	Assessment of English language anxiety and its relationship with academic achievement	English language anxiety	Academic achievement
(Fauzi and Asi, 2023)	Indonesia	Case study	University students	EFL learners	Examination of speaking anxiety among Indonesian university learners	Speaking anxiety	Sources of anxiety and communication challenges
(Cedar and Jaiyote, 2024)	Thailand	Cross-sectional comparative study	University students (2nd and 4th-year English majors)	EFL learners	Comparison of English-speaking anxiety across academic years	English-speaking anxiety	Differences by academic level and anxiety dimensions
(Demirdöken and Okur, 2023)	Turkey	Psychometric validation study with mediation analysis	Secondary and/or university students*	EFL learners	Development and validation of a speaking anxiety scale; mediation modeling	Speaking anxiety	Relationships among psychological and educational variables
(Huda and Khulel, 2025)	Indonesia	Cross-sectional survey	High school students	EFL learners	Investigation of psychological determinants and coping strategies related to English language anxiety	English language anxiety	Psychological determinants; coping strategies
(Rahmadani and Etfita, 2022)	Indonesia	Cross-sectional survey	University students	EFL learners	Assessment of foreign language speaking anxiety in English learning	Foreign language speaking anxiety	Factors associated with anxiety and speaking performance
(Achanan et al., 2021)	Malaysia	Cross-sectional survey	TESL undergraduate students	ESL learners	Examination of second-language speaking anxiety among future English teachers	Second-language speaking anxiety	Anxiety dimensions and communication challenges
(Hakim, 2022)	Indonesia	Cross-sectional study	University students	EFL learners	Analysis of foreign language anxiety according to gender and speaking performance in online learning environments	Foreign language anxiety	Gender differences: speaking performance
(Ratha and Shah, 2018)	Malaysia	Cross-sectional survey	Secondary school students	ESL learners	Examination of anxiety and attitudes toward English language learning	English language anxiety and learning attitudes	Relationship between anxiety and attitudes toward English
(Pohan and Kusumawardany, 2023)	Indonesia	Cross-sectional survey	University students	EFL learners	Exploration of students’ perceptions of speaking anxiety from a psychological perspective	Speaking anxiety perceptions	Psychological factors associated with anxiety
(Katemba, 2013)	Indonesia	Cross-sectional correlational study	Secondary school students	EFL learners	Assessment of anxiety levels and their relationship with English academic achievement	English language anxiety	Academic achievement in English
(Sajjad et al., 2022)	Pakistan	Cross-sectional survey	BS-level university students	ESL/EFL learners	Examination of the prevalence of English-speaking anxiety among university students	English-speaking anxiety	Anxiety prevalence and contributing factors
(Daflizar, 2024)	Indonesia	Cross-sectional correlational study	University students	EFL learners	Investigation of out-of-class speaking anxiety and its associations with learner characteristics	Out-of-class English-speaking anxiety	Self-perceived speaking skills, vocabulary proficiency, and gender
(Aditya et al., 2024)	Indonesia	Cross-sectional correlational study	Grade 11 high school students	EFL learners	Examination of the relationship between anxiety level and English-speaking competency	English-speaking anxiety and speaking competency	Speaking performance/competency relationship
(Tristeza and Tristeza, 2021)	Philippines	Cross-sectional survey	University/tertiary-level EFL learners	EFL learners	Exploration of attitudes toward English and speaking anxiety among learners	Speaking anxiety and attitudes toward English	Relationship between attitude and anxiety
(Aarif et al., 2019)	Malaysia	Cross-sectional survey	Polytechnic students	ESL learners	Investigation of speaking anxiety in communicative English classrooms	Speaking anxiety	Classroom communication challenges
(Erza and Ivan, 2025)	Indonesia	Cross-sectional case study	University students (7th semester)	EFL learners	Examination of anxiety in English-speaking classes among senior university students	English-speaking class anxiety	Contributing psychological and classroom factors
(Tridinanti, 2018)	Indonesia	Cross-sectional correlational study	Undergraduate students	EFL learners	Examination of relationships among speaking anxiety, self-confidence, and speaking achievement	Speaking anxiety and speaking achievement	Self-confidence; speaking performance
(Shanmugan et al., 2025)	Malaysia	Cross-sectional survey	Primary school students (Tamil vernacular schools)	ESL learners	Assessment of English speaking anxiety among young learners in vernacular school settings	English-speaking anxiety	Prevalence and contributing classroom factors
(Karakaya and Küçüktepe, 2023)	Turkey	Cross-sectional comparative study	Vocational and technical high school students	EFL learners	Investigation of English-speaking anxiety across vocational/technical school contexts	English-speaking anxiety	Differences across school types and learning contexts
(Idrus, 2021)	Malaysia	Case study	Secondary school students (rural areas)	EFL learners	Profiling English language learning anxiety in a rural school context	English language learning anxiety	Anxiety patterns and contributing factors
(Özcanlı and Kozikoğlu, 2024)	Turkey	Cross-sectional correlational study	Secondary school students	EFL learners	Examination of relationships among speaking anxiety, self-efficacy, and personality traits	English-speaking anxiety	Self-efficacy, personality traits

### ROBINS-I assessment

**Figure 2:**
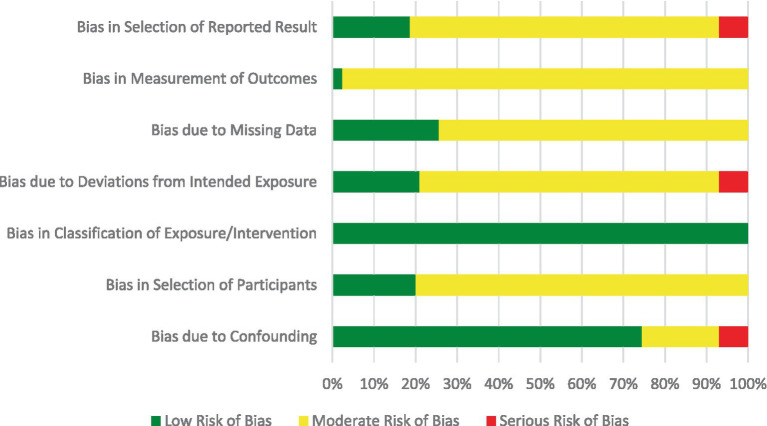
ROBINS-I assessment graph.

In the examined studies, there is a predominant presence of moderate risk of bias based on the ROBINS-I assessment. In contrast, the number of studies assessed as having low or low-to-moderate risk of bias is small. Most of the studies exhibited a moderate risk of bias in confounding and participant selection due to a tendency to use convenience samples and cross-sectional study designs in studies of EFL anxiety. The classification of exposure resulted in a low risk of bias across all studies, as all measurements of interventions or constructs were consistent. Measurement bias was moderate since it involved self-reported anxiety and speaking performance scales. Fewer studies demonstrated a low risk of bias owing to the use of experimental or control study designs. Some other studies were assessed as having a serious risk of bias due to high confounding and deviations from the exposure (Figure 2), (Table 3).

**Table 3:** ROBINS-I assessment of the studies.

Study	D1	D2	D3	D4	D5	D6	D7
Aarif et al. (2019)	?	?	+	?	+	?	?
Achanan et al. (2021)	−	?	+	−	?	?	−
Aditya et al. (2024)	?	+	+	+	+	?	+
Badriyah and Novita (2023)	+	?	+	?	+	?	+
Cedar and Jaiyote (2024)	?	?	+	?	+	?	?
Chen et al. (2022)	+	+	+	+	+	?	+
Daflizar (2024)	?	?	+	?	?	+	?
Demirdöken and Okur (2023)	+	+	+	+	+	+	+
Erza and Ivan (2025)	+	?	+	+	+	+	+
Fauzi and Asi (2023)	+	?	+	+	+	+	+
Mayer et al. (2014)	+	+	+	+	+	?	+
Miskam and Saidalvi (2019)	+	?	+	+	?	?	+
Özcanlı and Kozikoğlu (2024)	+	+	+	?	+	?	+
Pohan and Kusumawardany (2023)	?	+	+	+	?	?	+
Puri et al. (2025)	+	+	+	+	+	?	+
Rahmadani and Etfita (2022)	?	+	+	?	?	+	?
Ratha and Shah (2018)	?	+	+	+	+	?	+
Said and Weda (2018)	?	+	+	−	?	?	−
Zamri and Hashim (2023)	+	?	+	+	+	+	+
Feng et al. (2025)	+	+	+	+	+	+	+
Hakim (2022)	+	?	+	?	+	+	+
Huda and Khulel (2025)	?	+	+	?	+	+	+
Idrus et al. (2021)	+	?	+	?	+	+	+
James et al. (2019)	−	?	+	−	?	+	−
Karakaya and Küçüktepe (2023)	?	+	+	?	+	+	?
Katemba et al. (2013)	+	?	+	+	?	+	?
Keny et al. (2026)	?	+	+	?	+	?	?
Li et al. (2022)	+	+	+	+	+	?	+
Lin et al. (2016)	+	+	+	+	+	?	+
Marpaung and Fithriani (2023)	?	+	+	?	+	?	+
Sajjad et al. (2022)	?	?	+	?	?	?	?
Shanmugan et al. (2025)	?	+	+	?	?	+	?
Sinadia and Ngingi (2023)	+	?	+	+	+	+	+
Tam et al. (2026)	+	+	+	+	+	?	+
Tamco et al. (2021)	?	?	+	?	+	?	?
Tridinanti (2018)	+	?	+	+	+	+	+
Tristeza and Tristeza (2021)	?	+	+	+	+	+	+

D1=bias due to confounding, D2=bias in selection of participants, D3=bias in classification of exposure/intervention, D4=bias due to deviations from intended exposure, D5=bias due to missing data, D6=bias in measurement of outcomes, D7=bias in selection of reported result.

D1 = Bias due to Confounding, D2 = Bias in Selection of Participants, D3 = Bias in Classification of Exposure/Intervention, D4 = Bias due to Deviations from Intended Exposure, D5 = Bias due to Missing Data, D6 = Bias in Measurement of Outcomes, D7 = Bias in Selection of Reported Result.

### GRADE assessment

The studies have been classified into five themes based on their design, population, and analysis. The first theme (multimedia learning, cognitive load, and AI-supported instruction) consists of experimental studies that were highly rigorous in their methodology and were therefore classified as GRADE A, indicating high certainty in learning outcomes and anxiety reduction. Theme 2 includes core survey-based studies that examine speaking anxiety, having consistent correlations but at moderate risk of bias, and therefore classified as GRADE B. Theme 3 includes psychological variables such as personality and self-efficacy, using the SEM model and hence having high predictive validity (B + grade). Groups 4 and 5 included contextual and vulnerable populations and had lower methodological rigor and GRADE C–D certainty (Table 4).

**Table 4:** GRADE assessment of the included studies.

Group (domain)	No. of studies	Study design	Population context	Method quality	Effect type	GRADE
Multimedia learning, cognitive load and AI-enhanced language learning	7	Experimental/quasi-experimental/AI-assisted learning	University, EFL learners, secondary students	High internal validity, controlled designs	Learning outcomes, anxiety reduction, and cognitive load	GRADE A
English speaking anxiety	20	Cross-sectional surveys/correlational designs	Primary, secondary, and university EFL learners	Moderate validity (self-report bias common)	Anxiety levels, correlates, predictors	GRADE B
Psychological models (personality, self-efficacy, structural relations)	10	Correlational + SEM + mediation models	Undergraduate & secondary learners	Strong statistical modeling but non-experimental	Predictive relationships, structural pathways	GRADE B+
Attitude, perception and classroom context studies	5	Descriptive surveys/thesis-based/qualitative mixed	School & rural learners	Moderate-to-low methodological rigor	Perceptions of anxiety	GRADE C
Context-specific and vulnerable population studies	5	Case studies/contextual surveys	Rural students, primary learners, and online classes	Low generalizability	Contextual anxiety patterns	GRADE C–D

### Leave-one-out sensitivity analysis

The sensitivity analysis of Leave One Out indicates that the combined effect size is very stable and independent of any single study. The results are highly consistent across all iterations and support the reliability, validity, and generalizability of the meta-analysis’s findings (Table 5).

**Table 5:** Leave-one-out sensitivity analysis table.

Omitted study	Pooled SMD	95% CI	Heterogeneity (*I*^**2**^)	Egger’s test (intercept, 95% CI, *t*, *p*)	Interpretation
No study omitted	0.52	0.47–0.57	10%	2.08 (−0.91 to 5.07), *t* = 1.365, *p* = 0.18	Stable effect
Aarif et al. (2019) omitted	0.53	0.48–0.58	11.7%	2.25 (−1.3 to 5.81), *t* = 1.244, *p* = 0.221	Stable effect
Achanan et al. (2021) omitted	0.54	0.49–0.58	8.2%	1.35 (−2.12 to 5.81), *t* = 0.761, *p* = 0.451	Stable effect
Aditya et al. (2024) omitted	0.53	0.48–0.58	12.4%	2.19 (−0.94 to 5.32), *t* = 1.37, *p* = 0.178	Stable effect
Badriyah and Novita (2023) omitted	0.53	0.48–0.57	11.1%	1.93 (−1.12 to 5.07), *t* = 1.24, *p* = 0.222	Stable effect
Cedar and Jaiyote (2024) omitted	0.53	0.48–0.58	12.3%	2.16 (−0.89 to 5.21), *t* = 1.385, *p* = 0.174	Stable effect
Chen et al. (2022) omitted	0.53	0.48–0.57	10.9%	2.00 (−1.01 to 5.01), *t* = 1.30, *p* = 0.201	Stable effect
Daflizar (2024) omitted	0.53	0.48–0.57	10.9%	2.00 (−1.01 to 5.01), *t* = 1.30, *p* = 0.201	Stable effect
Demirdöken and Okur (2023) omitted	0.53	0.48–0.58	12.4%	2.09 (−0.93 to 5.12), *t* = 1.355, *p* = 0.183	Stable effect
Erza and Ivan (2025) omitted	0.53	0.48–0.58	12.3%	2.08 (−0.97 to 5.13), *t* = 1.338, *p* = 0.188	Stable effect
Fauzi and Asi (2023) omitted	0.53	0.48–0.58	12.4%	2.17 (−0.9 to 5.23), *t* = 1.384, *p* = 0.174	Stable effect
Feng et al. (2025) omitted	0.53	0.48–0.58	12.4%	2.08 (−0.95 to 5.10), *t* = 1.346, *p* = 0.186	Stable effect
Hakim (2022) omitted	0.53	0.48–0.58	12.4%	2.11 (−0.93 to 5.15), *t* = 1.362, *p* = 0.181	Stable effect
Huda and Khulel (2025) omitted	0.53	0.48–0.58	12.4%	2.09 (−0.94 to 5.11), *t* = 1.351, *p* = 0.184	Stable effect
Idrus (2021) omitted	0.53	0.48–0.58	12.4%	1.97 (−1.12 to 5.07), *t* = 1.25, *p* = 0.219	Stable effect
James et al. (2019) omitted	0.54	0.49–0.58	12.6%	1.75 (−1.10 to 4.59), *t* = 1.203, *p* = 0.236	Stable effect
Karakaya and Küçüktepe (2023) omitted	0.53	0.48–0.57	11.4%	2.01 (−1.02 to 5.03), *t* = 1.301, *p* = 0.201	Stable effect
Katemba (2013) omitted	0.53	0.48–0.58	12.1%	2.06 (−0.96 to 5.08), *t* = 1.335, *p* = 0.189	Stable effect
Keny et al. (2026) omitted	0.53	0.48–0.58	12.3%	2.08 (−0.94 to 5.11), *t* = 1.349, *p* = 0.185	Stable effect
Li et al. (2022) omitted	0.53	0.48–0.57	11.5%	1.98 (−1.07 to 5.03), *t* = 1.271, *p* = 0.211	Stable effect
Lin et al. (2016) omitted	0.53	0.48–0.58	12%	2.22 (−0.82 to 5.25), *t* = 1.429, *p* = 0.161	Stable effect
Marpaung and Fithriani (2023) omitted	0.53	0.48–0.58	12.3%	2.44 (−0.75 to 5.63), *t* = 1.498, *p* = 0.142	Stable effect
Mayer et al. (2014) omitted	0.53	0.48–0.57	11.4%	2.16 (−0.84 to 5.17), *t* = 1.41, *p* = 0.166	Stable effect
Miskam and Saidalvi (2019) omitted	0.53	0.48–0.58	12.1%	2.06 (−0.96 to 5.08), *t* = 1.335, *p* = 0.189	Stable effect
Özcanlı and Kozikoğlu (2024) omitted	0.53	0.48–0.58	12.0%	2.22 (−0.82 to 5.25), *t* = 1.429, *p* = 0.161	Stable effect
Pohan and Kusumawardany (2023) omitted	0.53	0.48–0.57	11.3%	2.11 (−0.89 to 5.12), *t* = 1.379, *p* = 0.176	Stable effect
Puri et al. (2025) omitted	0.53	0.48–0.58	12.1%	2.07 (−0.95 to 5.09), *t* = 1.344, *p* = 0.187	Stable effect
Rahmadani and Etfita (2022) omitted	0.53	0.48–0.58	12.1%	2.05 (−0.98 to 5.08), *t* = 1.324, *p* = 0.193	Stable effect
Ratha and shah (2018) omitted	0.53	0.48–0.58	12.1%	2.08 (−0.94 to 5.10), *t* = 1.349, *p* = 0.185	Stable effect
Said and Weda (2018) omitted	0.53	0.48–0.58	12.0%	2.14 (−0.89 to 5.16), *t* = 1.385, *p* = 0.174	Stable effect
Sajjad et al. (2022) omitted	0.53	0.48–0.58	12.0%	2.10 (−0.91 to 5.12), *t* = 1.366, *p* = 0.180	Stable effect
Shanmugan et al. (2025) omitted	0.54	0.49–0.58	12.2%	2.01 (−0.86 to 4.89), *t* = 1.373, *p* = 0.177	Stable effect
Sinadia and Ngingi (2023) omitted	0.53	0.48–0.58	12.1%	2.05 (−0.98 to 5.08), *t* = 1.324, *p* = 0.193	Stable effect
Tam (2026) omitted	0.53	0.48–0.58	12%	2.14 (−0.89 to 5.16), *t* = 1.385, *p* = 0.174	Stable effect
Tamco (2021) omitted	0.53	0.48–0.58	12.3%	2.16 (−0.89 to 5.21), *t* = 1.385, *p* = 0.174	Stable effect
Tridinanti (2018) omitted	0.53	0.48–0.57	12.2%	2.07 (−0.94 to 5.08), *t* = 1.35, *p* = 0.185	Stable effect
Tristeza and Tristeza (2021) omitted	0.53	0.48–0.58	12.1%	2.05 (−0.98 to 5.08), *t* = 1.324, *p* = 0.193	Stable effect
Zamri and Hashim (2023) omitted	0.54	0.50–0.59	12.3%	2.59 (0.11 to 5.07), *t* = 2.045, *p* = 0.048	Stable effect

### Overall meta-analytic findings across domains

In 38 studies, all domains yielded statistically significant benefits in a random-effects model analysis. The pooled SMD values ranged from 0.44 to 0.54 (*p* < 0.05). This indicates small to moderate effect sizes. SMD for multimedia learning is 0.44 (*I*^2^ = 45.6%), SMD for anxiety reduction programs is 0.51 (*I*^2^ = 13.5%), SMD for academic achievement and learner characteristics is 0.52 (*I*^2^ = 27.5%), and SMD for speaking anxiety in various contexts is 0.54 (*I*^2^ = 16.7%). The heterogeneity is low to moderate in this case, implying consistency in results. Egger’s test for all domains was not significant (*p* > 0.05), and funnel plots indicate no publication bias (Table 6).

**Table 6:** Overall meta-analytic findings across domains.

Domain	No. of studies	Pooled effect size (SMD)	95% CI	Overall effect (*p*-value)	τ^**2**^	*I*^**2**^ (%)	Heterogeneity *p*-value	Egger’s intercept	95% CI of intercept	*t*-value	Egger’s *p*-value
Multimedia and technology-enhanced language learning	7	0.44	0.31–0.58	<0.05	0.0103	45.6	0.09	−2.21	−7.34 to 2.93	−0.842	0.438
Anxiety reduction and cognitive-affective interventions	8	0.52	0.40–0.62	<0.05	0.0049	13.5	Not significant	6.07	−2.99 to 15.12	1.314	0.221
Anxiety, academic performance, and learner characteristics	9	0.51	0.41–0.62	<0.05	0.0077	27.5	Not significant	3.44	−5.45 to 12.33	0.758	0.470
Speaking anxiety across educational contexts	19	0.54	0.45–0.61	<0.05	0.0029	16.7	Not significant	−0.22	−10.81 to 10.38	−0.04	0.968

### Group analysis

#### Group 1. Multimedia and technology-enhanced language learning

**Figure 3:**
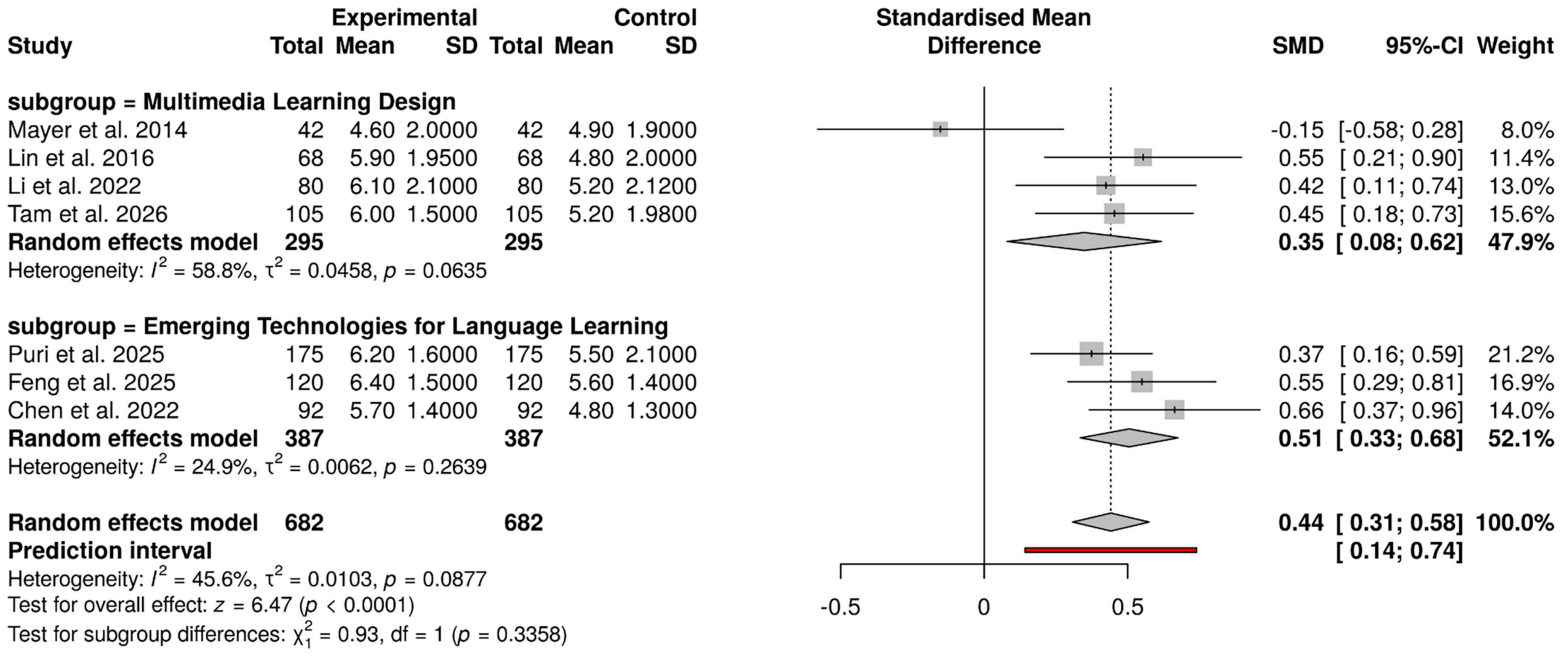
Forest plot of the studies about multimedia and technology-enhanced language learning.

In total, 7 studies were examined using a random-effects model. The meta-analysis revealed a significant advantage for the experimental group in terms of effect size (SMD = 0.44, 95% CI: 0.31–0.58, *p* < 0.05), indicating a small-to-moderate effect of multimedia and technology-enhanced language learning. In particular, all subgroups regarding multimedia design and new technologies (AI, mobile learning, and AR) consistently indicated an advantage for the interventions. Heterogeneity of the findings was moderate (*I*^2^ = 45.6%, τ^2^ = 0.0103, *p* = 0.09). There was no evidence of publication bias (Egger’s test *p* = 0.438), (Figure 3).

#### Group 2. Anxiety reduction and cognitive-affective interventions

**Figure 4:**
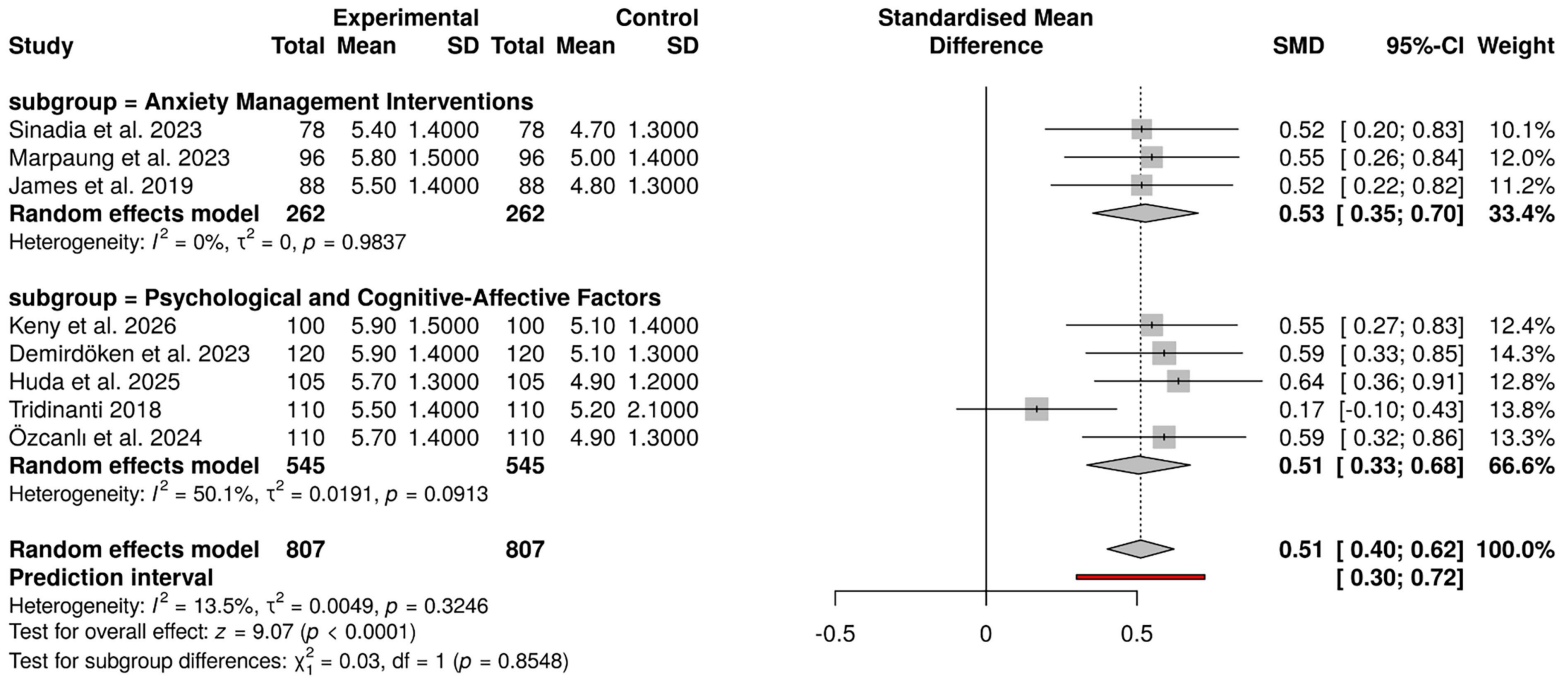
Forest plot of the studies about anxiety reduction and cognitive-affective interventions.

Eight studies were selected for the meta-analysis and analyzed using the random-effects model. The overall effect size for the decrease in language anxiety was significant (SMD = 0.51, 95% CI: 0.40–0.62, *p* < 0.05). Thus, a moderate positive impact of anxiety reduction and cognitive-affective interventions can be assumed. Subgroup 2A indicated that anxiety management interventions increased confidence in speaking and emotional regulation. In contrast, subgroup 2B stressed the importance of self-efficacy, personality factors, and coping mechanisms in terms of anxiety reduction. There was low heterogeneity in results (*I*^2^ = 13.5%, τ^2^ = 0.0049). No publication bias was detected (Egger’s test *p* = 0.221), (Figure 4).

#### Group 3. Anxiety, academic performance, and learner characteristics

**Figure 5:**
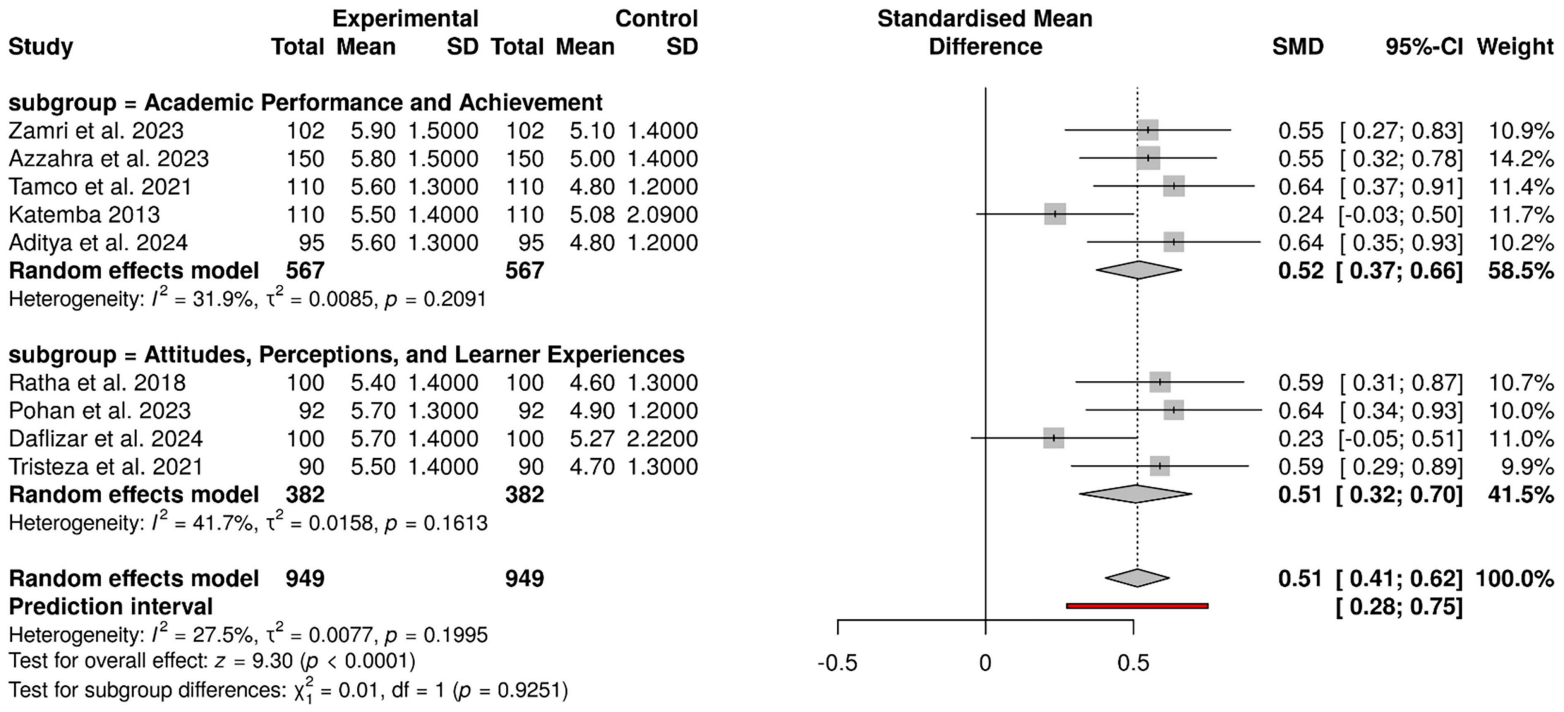
Forest plot of the studies about anxiety, academic performance, and learner characteristics.

Overall, 9 studies were included in the random-effects model. The pooled effect indicated a statistically significant relationship between anxiety, academic achievement, and learner characteristics (SMD = 0.51, 95% CI: 0.41–0.62, *p* < 0.05). Subgroup 3A found that low anxiety level was associated with high academic achievement and proficiency in the English language. In contrast, subgroup 3B showed the role of attitude, perceptions, and experience of learners in terms of causing anxiety. There was no evidence of heterogeneity (*I*^2^ = 27.5%, τ^2^ = 0.0077), which shows consistency among different studies (Figure 5).

#### Group 4. Speaking anxiety across educational contexts

**Figure 6:**
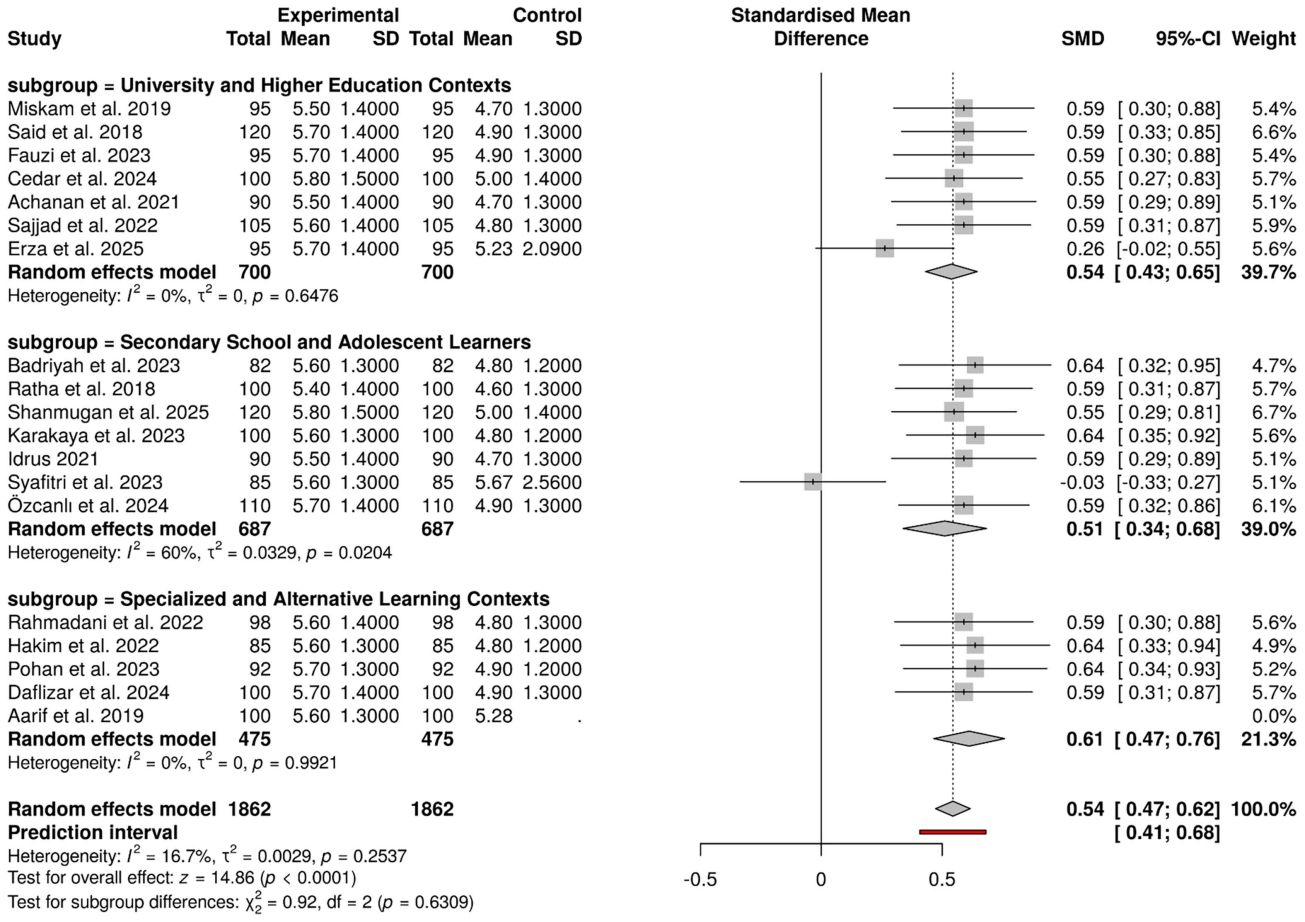
Forest plot of the studies about speaking anxiety across educational contexts.

In all, 19 studies underwent meta-analysis using random-effects models. Pooled data indicated a statistically significant decrease in speaking anxiety across all educational settings (SMD = 0.54, 95% CI: 0.47–0.62, *p* < 0.05), indicating a moderate and consistent effect. Sub-group 4A had high anxiety levels of university and higher education learners, whereas Sub-group 4B experienced anxiety in the same manner as secondary and adolescent students. Sub-group 4C had anxiety levels in specialized educational environments, such as online and vocational learning institutions. The findings have low heterogeneity (*I*^2^ = 16.7%, τ^2^ = 0.0029). There was no evidence of publication bias (Egger’s test *p* = 0.968), (Figure 6).

### Publication bias assessment

**Figure 7:**
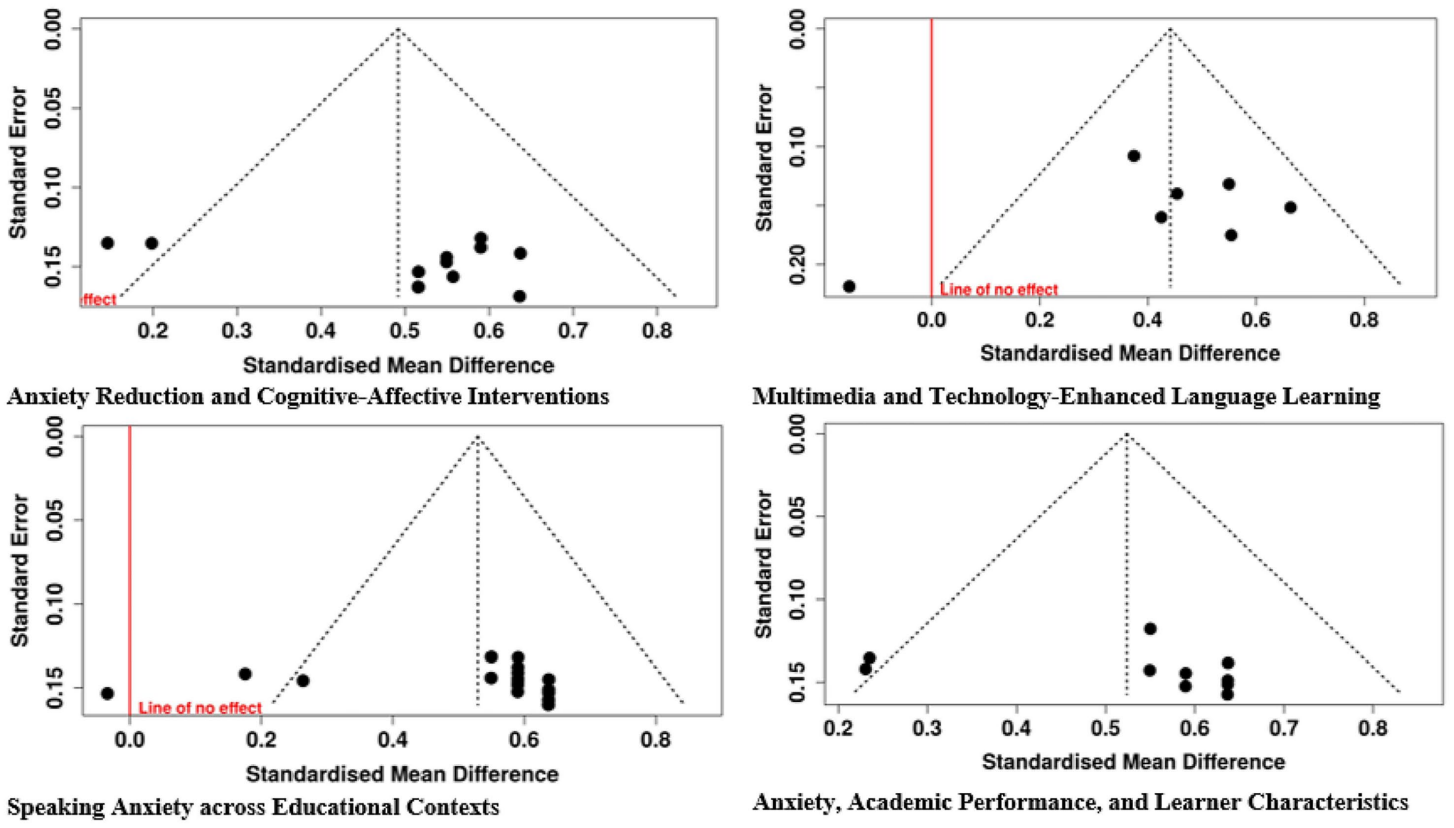
Funnel plot of the included studies.

Egger’s regression test was performed to assess funnel plot asymmetry. If the *p*-value obtained was below 0.05, this signified publication bias. In all four areas analyzed, there was no significant publication bias, as evidenced by the Egger’s test results. Particularly in the area of multimedia and technology-enhanced language learning, there was an Egger’s intercept value of −2.21 (95% CI: −7.34 to 2.93; *p* = 0.438), while in the area of anxiety reduction and cognitive-affective interventions, the intercept value was 6.07 (95% CI: −2.99 to 15.12; *p* = 0.221). The intercept value in the area of anxiety and academic performance was 3.44 (95% CI: −5.45 to 12.33; *p* = 0.470), whereas in the area of speaking anxiety in various educational contexts, it was −0.22 (95% CI: −10.81 to 10.38; *p* = 0.968), (Figure 7).

## Discussion

The data from 38 research investigations on multimedia-assisted language acquisition, cognitive load, speaking anxiety, and learner-related factors in EFL/ESL situations was combined in this systematic review and meta-analysis. The results imply that the efficiency of technology-enhanced instruction depends on how multimedia learning environments are created to accommodate learners’ limited working-memory capacity rather than showing a consistent benefit. The Cognitive Load Theory and the Cognitive Theory of Multimedia Learning, which suggest that good instructional design reduces needless cognitive processing while promoting the integration of verbal and visual information, are in line with this approach (Li et al., 2022; Lin et al., 2016; Mayer et al., 2014; Puri et al., 2025; Feng, 2025).

### Multimedia and technology-enhanced language learning

The results show that multimedia and technology-enhanced training can aid language learning when digital materials are created to reduce needless cognitive processing and promote efficient information presentation. Multimedia technologies are most helpful when they facilitate effective processing of verbal and visual information, according to studies using captions, e-notes, multimodal input, mobile learning, AI-supported instruction, and augmented reality (Li et al., 2022; Lin et al., 2016; Mayer et al., 2014; Puri et al., 2025; Feng, 2025; Chen et al., 2022; Tam, 2026). According to CLT, these interventions may lessen unnecessary cognitive burden by enhancing the synchronisation of visual, auditory, and textual information. From the standpoint of CTML, they might aid in the integration of verbal and visual channels, which would enhance learning.

It is not appropriate to consider the somewhat lesser effect found in the multimedia realm as poor evidence. The moderate pooled effect may be explained by differences in instructional design, learner proficiency, task type, modality, tempo, and feedback among multimedia learning settings. In line with CLT, poorly integrated multimedia resources could actually make learning more difficult rather than easier. Consequently, the results indicate that multimedia-assisted language acquisition works best when instructional design minimises superfluous processing and promotes fruitful cognitive engagement.

### Cognitive-affective interventions and speaking anxiety

The beneficial effects of cognitive-affective and anxiety-reduction therapies imply a close relationship between emotional and cognitive processes during language acquisition. The cognitive demands of language learning may be increased by speaking anxiety and associated psychological characteristics, such as self-efficacy and self-confidence, which would reduce the cognitive resources available for oral performance and language comprehension (Simanungkalit and Likuayang, 2023; Puri et al., 2025; Sinadia and Ngingi, 2023; Marpaung and Fithriani, 2023). As a result, anxiety-reduction strategies may promote more effective cognitive processing when doing language-learning tasks.

This explanation is in line with the notable impacts of speaking anxiety that have been reported in several educational contexts. Speaking exercises add to intrinsic cognitive load from a CLT perspective, but speaking anxiety may raise the total cognitive demands encountered during language learning. Self-confidence, self-efficacy, personality, learner views, and coping mechanisms were found to be connected with speaking anxiety and language-learning outcomes in the included research (Sinadia and Ngingi, 2023; Marpaung and Fithriani, 2023). All of these results point to speaking anxiety as a significant cognitive-affective factor that affects language acquisition.

### Learner characteristics and academic performance

Both instructional and learner-related features may have an impact on language-learning outcomes, as indicated by the strong correlation between learner-related parameters and academic performance. Learner competence, self-efficacy, confidence, attitudes, and learner perceptions were consistently linked to academic success and language-learning outcomes in the included research (Lin et al., 2016; Chen et al., 2022; James et al., 2019; Zamri and Hashim, 2023; Miskam and Saidalvi, 2019). These results suggest that students may react differently to speaking and multimedia assignments depending on their unique qualities and learning environment. The substantial pooled effect shown for learner attributes and academic performance is consistent with this explanation. The results collectively imply that learner-related variables that affect engagement, confidence, and language performance are just as important to successful multimedia-assisted language acquisition as proper instructional design.

### Subjective and objective cognitive load measures

The included research evaluated cognitive burden mainly by subjective measures, such as self-report instruments and standardised cognitive load scales in addition to performance-based outcomes like comprehension, recall, and vocabulary acquisition. Furthermore, some research used objective methods like electroencephalography to quantify cognitive load during multimedia activities in real time, offering additional insights into cognitive processes (Simanungkalit and Likuayang, 2023). Overall, relatively few research included objective physiological measurements, and the majority of the information compiled in this review came from subjective evaluations.

### Interpretation of heterogeneous findings

Pooled effects were statistically significant in all four domains, however the degree of heterogeneity and the size of the impacts varied depending on the analysis. Multimedia and technology-enhanced language learning demonstrated moderate heterogeneity (*I*^2^ = 45.6%), while anxiety-reduction and cognitive-affective interventions (*I*^2^ = 13.5%), learner characteristics and academic performance (*I*^2^ = 27.5%), and speaking anxiety across educational contexts (*I*^2^ = 16.7%) showed less heterogeneity. Subtitles, captions, e-notes, mobile learning, AI-supported learning, augmented reality, and multimodal feedback are only a few of the multimedia educational methodologies covered in the review, which may account for this heterogeneity (Li et al., 2022; Lin et al., 2016; Mayer et al., 2014; Puri et al., 2025; Feng, 2025; Chen et al., 2022; Tam, 2026).

Symmetric funnel plots and non-significant Egger’s regression tests showed no indication of publication bias in any domain. Furthermore, the leave-one-out sensitivity analysis demonstrated that the pooled effect sizes stayed constant after individual studies were sequentially excluded, suggesting that no single study had a disproportionate impact on the overall findings.

## Data Availability

The raw data supporting the conclusions of this article will be made available by the authors, without undue reservation.
